# Organerhalt nach Short-course-Radiotherapie und transanaler endoskopischer Mikrochirurgie bei T1-2-Rektumkarzinomen

**DOI:** 10.1007/s00066-021-01795-0

**Published:** 2021-06-04

**Authors:** Julius Pochhammer, Jürgen Dunst

**Affiliations:** 1 Chirurgische Universitätsklinik, UKSH, Campus Kiel, Arnold-Heller-Str. 3, 24105 Kiel, Deutschland; 2 Klinik für Strahlentherapie, UKSH, Campus Kiel, Arnold-Heller-Str. 3, 24105 Kiel, Deutschland

## Hintergrund

Die totale mesorektale Exzision (TME) ist sehr erfolgreich hinsichtlich lokaler Tumorkontrolle beim Rektumkarzinom, aber sie ist, insbesondere bei Lokalisation des Tumors im unteren und mittleren Drittel, mit einem substanziellen Risiko für Funktionsverlust und Spätfolgen verbunden. Offensichtlich ist dies bei Rektumexstirpation mit Anus praeter, aber auch nach sphinktererhaltender Operation treten Langzeitfolgen bezüglich Kontinenz und Sexualfunktion auf. Mit zunehmender onkologischer Sicherheit ist dieses Thema in den letzten Jahren stärker in den Fokus gerückt, und seit mehreren Jahren gibt es erste Bestrebungen für einen Organerhalt auch beim tiefsitzenden Rektumkarzinom [5, 7].

Bei fortgeschrittenen Tumoren der Kategorien T3‑4 wird durch die präoperative Radiochemotherapie (RCT) in etwa 15 % eine komplette Remission (CR) erreicht. Mehrere kleinere Studien deuten darauf hin, dass die Mehrzahl dieser Patienten (geschätzt etwa 70 %) auch ohne Operation dauerhaft lokal tumorfrei bleibt. Daher ist nach der aktuellen Leitlinie bei Patienten mit klinischer Vollremission nach neoadjuvanter RCT ein abwartendes Vorgehen mit dem Ziel eines Rektumerhalts erlaubt. Durch totale neoadjuvante Therapie (TNT) wird die CR-Rate erhöht; das ist das wesentliche Argument für die TNT.

Frühe Stadien des Rektumkarzinoms (also die Kategorien T1-2) werden bisher ohne neoadjuvante Radio(chem)therapie primär operiert; in Großbritannien erfolgen circa 25 % der TME wegen T1- oder T2-Tumoren [2]. Daraus resultiert eine gewisse Diskrepanz: Patienten mit T3-Tumoren haben eine bessere Chance auf Organerhalt als Patienten mit T2-Tumoren. Es ist daher logisch, eine organerhaltende Strategie durch Radiochemotherapie auch bei T1-2-Karzinomen zu prüfen. Es gibt seit mehr als zehn Jahren eine Reihe prospektiver einarmiger Studien, die ein solches Konzept möglich erscheinen lassen, z. B. die ACOSOG-Studie mit 79 Patienten [3], aber keine randomisierten Studien. In der kürzlich publizierten TREC-Studie wurde geprüft, ob eine Randomisierung zu dieser Fragestellung durchführbar ist und von den Patienten akzeptiert wird [1].

## Patienten und Methodik

TREC war eine randomisierte Studie an 21 britischen spezialisierten Krankenhäusern („tertiary hospitals“, entspricht in Deutschland der Schwerpunktversorgung). Eingeschlossen wurden Patienten mit T1‑2 Rektumkarzinomen (max. Durchmesser 3 cm). Die Patienten wurden randomisiert und entweder mit TME oder mit Kurzzeitvorbestrahlung und (nach 8–01 10 Wochen) einer transanalen endoskopischen Mikrochirurgie mit angestrebtem Organerhalt unterzogen. In dieser Gruppe wurde eine TME bei histologischen Risikofaktoren nach organerhaltender Op. empfohlen. Primärer Endpunkt war die Randomisierungsrate. Patienten, die für eine Randomisierung nicht geeignet erschienen, wurden als nichtrandomisierte Kontrolle beobachtet.

## Ergebnisse

Zwischen 2012 und 2014 wurden 55 Patienten an 15 Prüfzentren randomisiert, 27 für ein organerhaltendes Vorgehen und 28 für die TME-Kontrollgruppe. Acht der 27 Patienten (30 %) mit Kurzzeitbestrahlung und endoskopischer Op. hatten eine komplette Remission, 16 (59 %) wiesen nach Op. histologische Risikofaktoren auf (bei primärer TME war dieser Anteil 86 %) und 8 (30 %) der vorbehandelten Patienten erhielten eine TME; ein Patient mit CR wurde nicht operiert. Langfristiger Organerhalt (also lediglich eine transanale Tumorresektion) wurde in 70 % der RT-Gruppe erreicht. Kein Patient starb innerhalb von 30 Tagen, 1 Patient der RT-Gruppe innerhalb von 6 Monaten. Drei Patienten der RT-Gruppe entwickelten ein Lokalrezidiv, das nur bei zwei Patienten erfolgreich operiert werden konnte; ein Patient war wegen kardiovaskulärer Risiken nicht operabel. Alle drei Patienten mit Lokalrezidiv hatten Risikofaktoren nach endoskopischer OP (2-mal ypT3, 1‑mal R1) und hatten die empfohlene sofortige TME abgelehnt. Schwere unerwünschte Ereignisse (SAE) traten bei primärer TME häufiger auf als bei Organerhalt (39 % versus 15 %). Das krankheitsfreie Überleben (DFS) nach 3 Jahren (85 % versus 76 %, *p* = 0,12) und das Gesamtüberleben (OS, 93 % versus 88 %, *p* = 0,37) waren nicht signifikant verschieden (aber jedoch besseren absoluten Werten nach primärer TME). Im Langzeit-Follow-up bestanden signifikante Verschlechterungen der Lebensqualität in der Gruppe mit primärer TME; das betraf die „overall QoL“, die Rollen- und Sexualfunktion und das körperliche Aussehen.

68 Patienten wurden als nichtrandomisierte Gruppe behandelt und beobachtet; 61 wurden mit primärer Organerhaltung behandelt, vorwiegend wegen Alter oder Komorbiditäten. Alle Zentren, die mindestens drei Patienten in die Studie einbrachten, hatten auch mindestens einen randomisierten Patienten.

## Schlussfolgerungen der Autoren

Die Autoren zeigen sich zufrieden mit der Tatsache, dass es offensichtlich möglich ist, eine multizentrische randomisierte Studie zur Frage des Organerhalts durchzuführen. Die TREC-Studie sei die erste randomisierte Studie, in der ein Vorteil eines primär organerhaltenden Vorgehens bezüglich Lebensqualität nachgewiesen wurde. Der nächste Schritt wäre eine große randomisierte Studie, die bereits gestartet wurde (STAR-TREC-Studie).

## Kommentar

Die hohe onkologische Sicherheit, die wir heute hinsichtlich lokoregionaler Tumorkontrolle auch beim Rektumkarzinom erreichen, erlaubt und verlangt, den Blick auf das Vermeiden von Nebenwirkungen zu richten. Dass wir heute ausgewählten Patienten mit Rektumkarzinom (nämlich denjenigen mit kompletter Remission nach RCT) eine nichtoperative Option anbieten können, hätte man vor 20 Jahren als Illusion abgetan. Es ist aber logisch, diesen Weg weiter zu gehen und die nichtoperative Sanierung der frühen Stadien zu prüfen. Die TREC-Studie ist ein weiterer Schritt in diese Richtung, aber man muss schon die Daten aktuell mit Vorsicht interpretieren:

1. Die Studie hatte einen ungewöhnlichen Endpunkt, nämlich die Randomisierungsquote. Das ist innovativ und durchdacht. Bevor man Zeit und Geld in eine große randomisierte Studie investiert, wollte man zunächst sicherstellen, dass ein solches Konzept flächendeckend durchführbar ist und angenommen wird. Das konnte hier nachgewiesen werden. Allerdings ist festzustellen, dass pro Prüfzentrum etwas mehr als ein Patient pro Jahr rekrutiert wurde. Da es sich in der Regel um high-volume Zentren handelt, spricht dies Frequenz nicht für eine hohe Akzeptanz.
2. Das präoperative Konzept (hier eine einfache und kurze Vorbestrahlung mit 5 × 5 Gy) ist diesem Design geschuldet. Es ist klar, dass diese Art der Strahlentherapie vermutlich nicht das Optimum darstellt, mit dem man stabile Remissionen erreicht. Daher wird in der randomisierten Folgestudie (STAR-TREC) neben der Kurzzeitvorbestrahlung auch eine Radiochemotherapie geprüft [6].
3. Die bisherigen Ergebnisse machen Hoffnung, dass ein organerhaltendes Konzept funktionieren könnte. Aber die Daten sind eben präliminär und berechtigen nicht zur Änderung der Leitlinien oder zu Abweichungen von ihnen. Andererseits gibt es sicherlich Situationen, in denen man ein organerhaltendes Konzept individuell diskutieren kann, z. B. bei älteren Patienten mit T1-2-Karzinomen und erheblich erhöhtem Op.-Risiko. Das spiegelt sich hier in der hohen Zahl von so behandelten Patienten in der nichtrandomisierten Gruppe wider.
4. Bezüglich des organerhaltenden Vorgehens muss jedoch etwas Wasser in den Wein gegossen werden. Zum einen sind in der Studie Patienten mit einem uT1-Stadium < 3 cm und ohne Lymphgefäßinvasion (*n* = 9 bzw. 10) laut deutschen S3-Leitlinien mit einer alleinigen transanalen Resektion ausreichend behandelt. Somit wurden diese in beiden Gruppen übertherapiert. Auch für uT2-Situationen ist eine transanale Resektion und lokale Kontrolle möglich. Allerdings besteht das Risiko in der Lymphknotenmetastasierung, die sich in der TME-Gruppe in ca. 15 % der Fälle findet und durch die Kurzzeitvorbestrahlung nicht ausreichend adressiert wird. Zieht man die übertherapierten Patienten (uT1) ab sowie die Patienten, denen eine TME empfohlen wurde, die die Patienten aber ablehnten, was später zum Rezidiv führte, resultiert eine deutlich niedrigere Rate an Organerhalt.
  Zum anderen sind auch nach Vorbestrahlung und transanaler Resektion die funktionellen Ergebnisse nicht mit dem prätherapeutischen Zustand vergleichbar. So besteht bei kompletter Remission nach neoadjuvanter RCT und „watch and wait“ bei ca. 30 % der Patienten das Vollbild eines „major LARS“ [8]. Die transanale Resektion verstärkt diesen Effekt; bis zu 80 % der Patienten haben danach milde oder ausgeprägte funktionelle Beschwerden [9].
5. Bezüglich der aufgeführten Lebensqualitätsdaten fällt auf, dass für die meisten Items bis zum 24. Monat kein Unterschied für die Population gezeigt werden konnte. Bis dahin waren in beiden Gruppen stets etwa 80 % der nachuntersuchten Patienten-LQ-Fragebögen existent. Für die Nachuntersuchung nach 36 Monaten lagen für die Gruppe mit Organerhalt nur noch Bögen für 68 % der zu diesem Zeitpunkt nachuntersuchten Patienten vor, und die Mittelwerte stiegen für diese Gruppe merklich an, sodass sich dann ein LQ-Vorteil ergab.
6. Die in der Studie aufgeführten Kaplan-Meier-Kurven weisen für die beiden Gruppen keinen signifikanten Unterschied bezüglich des OS und DFS auf. Da die Studie jedoch nicht für diese Endpunkte gepowert war, kann hierbei kein Rückschluss auf onkologische Ergebnisse gezogen werden, zumal sich die Kurven zugunsten der TME unterscheiden.
7. Für die Radioonkologen: Die Strahlentherapie bei T1‑2 erfordert natürlich adaptierte Zielvolumenkonzepte, und möglicherweise profitiert dieses Kollektiv in besonderem Maße von modernen Technologien der Strahlentherapie [4]. Diese Frage müssen wir im Lauf der nächsten Jahre klären.
8. Ein aus unserer Sicht enorm wichtiger Aspekt eines organerhaltenden Konzepts wird von den Autoren nur in einem Satz im Ergebnisteil der Publikation erwähnt: Es traten drei Lokalrezidive bei den primär organerhaltend behandelten Patienten auf, und alle drei Patienten hatten nach RT und transanaler Chirurgie die danach eigentlich wegen histologischer Risikofaktoren empfohlene TME abgelehnt. Diese Rezidive hätten also eigentlich problemlos vermieden werden können, sind damit aber nicht unter erreichtem Organerhalt zu summieren. Das zeigt auch: Nicht alle Patienten sind für eine Watch-and-wait-Strategie nach kompletter Remission geeignet, und die Vorauswahl und die anschließende Führung und Betreuung solcher Patienten sind anspruchsvolle interdisziplinäre Aufgaben.

## Fazit

Ein organerhaltendes Konzept für frühe Tumoren im unteren Rektum (insbesondere T2-Tumoren) ist wissenschaftlich und aus Patientensicht attraktiv. Weitere Studien laufen, sind aber auch nötig. Außerhalb von Studien ist ein solches Vorgehen zunächst allenfalls bei massiven Risikofaktoren gegen eine TME vertretbar. Eine Aufnahme in das Armamentarium der Behandlungsoptionen erscheint für die Zukunft wünschenswert.

*Julius Pochhammer und Jürgen Dunst, Kiel*
